# Effects of Family‐Centered Empowerment Model vs. Traditional Model on Adherence and Perceived Social Support in Bariatric Surgery Patients: ‎A Randomized Clinical Controlled Trial

**DOI:** 10.1002/hsr2.71407

**Published:** 2025-10-24

**Authors:** Mahboobeh Hosseinimoghadam, Sina Ghanbarzadeh, Zahra Sobhani, Masood Amini, Amirali Alizadeh, Zinat Mohebbi

**Affiliations:** ^1^ School of Nursing and Midwifery Shiraz University of Medical Sciences Shiraz Iran; ^2^ Student Research Committee, School of Medicine Shiraz University of Medical Sciences Shiraz Iran; ^3^ Colorectal Research Center Shiraz University of Medical Sciences Shiraz Iran; ^4^ Laparoscopy Research Center Shiraz University of Medical Sciences Shiraz Iran; ^5^ Student Committee of Medica, Education Development Center Maragheh University of Sciences Maragheh Iran

**Keywords:** adherence, bariatric surgery, educational methods, Family Centered Empowerment Model (FCEM), social support

## Abstract

**Background and Aims:**

Bariatric surgery is presently considered the optimal treatment option for reducing mortality and morbidity among individuals with obesity and educating and empowering patients and their families leads to better patient outcomes and increased participation in healthcare programs Thus, aim of this study was to evaluate the effects of two educational methods on adherence and perceived social support in bariatric surgery patients: the family‐centered empowerment model versus the traditional model.

**Methods:**

This randomized controlled trial study was conducted on 30 bariatric surgery patients referred to one of the hospitals of Shiraz University of Medical Sciences, and were randomly divided into two groups. A family‐centered intervention was performed for the intervention group (*n* = 15), and traditional model was provided to the control group (*n* = 15). A demographic questionnaire, the Bariatric Surgery Self‐Management Behaviors Questionnaire (BSSQ), the General Adherence Scale (GAS), and the Specific Adherence Scale (SAS), as well as the Multidimensional Scale of Perceived Social Support Questionnaire (MSPSS), were used to collect the data. Data were analyzed using SPSS software, version 21, with a significance level of 0.05.

**Results:**

The results of our study showed that there was no statistically significant difference in Self‐management behavior (*p* = 0.255), and General Adherence (*p* = 0.170), between the two groups after the intervention, but there was a significant difference in total Perceived Social Support (*p* = 0.015) and tow subscales; family (*p* = 0.006), and friends' support (*p* = 0.037) between the two groups after the intervention.

**Conclusion:**

Family‐centered models provide an opportunity to empower family members to become active participants in the patient's health. The model could provide an opportunity for the patients to develop health their habits, such as physical activity, which would help to maintain their weight loss.

**Trial Registration:**

IRCT, IRCT20180523039802N3. Registered December 13, 2020.

**Abbreviations**
BSSQ: bariatric surgery self‐management behaviors questionnaire
CFI: confirmatory fit index
CHOL: total cholesterol
DSM: dumping syndrome management
EB: eating behaviors
FBS: fasting blood sugar
FCEM: family centered empowerment model
FI: fluid intake
FVW: fruit, vegetable, and whole grain intake
GAS: General Adherence Scale
HC: hip circumference
HDL: high‐density lipoprotein
LDL: low‐density lipoprotein
LDQ: lifestyle distress questionnaire
MSPSS: multidimensional scale of perceived social support
PA: physical activity
PBQ: perceived benefits questionnaire
PI: protein intake
RMSEA: root mean square error of approximation
SAS: Specific Adherence Scale
SD: standard deviation
SI: supplement intake
TLI: Tucker‐Lewis Index
WC: waist circumference
WL: weight loss
WRSM: weight‐related symptom measure

## 1 Introduction

The prevalence of obesity across the globe has increased exponentially over the last few decades [1]. The number of obese adults is estimated to reach 1.12 billion by 2030 [2]. The World Health Organization (WHO) defines obesity as abnormal or excessive fat accumulation that poses a risk to a person's health. Several health problems including Type II diabetes, cardiovascular disease, cancer, psychological problems, and other health problems arise from these two conditions [3]. As a result, effective treatments, such as bariatric surgery, are needed to reduce the negative effects of obesity on patients [4]. Bariatric surgery is presently considered the optimal treatment option for reducing mortality and morbidity among individuals with obesity, specifically those with body mass indexes (BMIs) of 40 kg/m^2^ or higher [5]. Despite various benefits of bariatric surgery, it can lead to nutrient deficiencies due to decreased intake, altered eating habits, food intolerance, gastrointestinal issues, and malabsorption [6]. Therefore, after bariatric surgery, patients must follow recommendations to avoid postoperative complications such as anemia, dumping syndrome, and osteoporosis. This includes attending follow‐up appointments, following dietary and exercise recommendations, and taking vitamins [7, 8]. It is imperative to comply with post‐operative guidelines, as noncompliance has been proven to be strongly associated with less favorable results and is a major contributing factor to unsuccessful outcomes [8]. Unfortunately, maintaining these habits can be challenging, and weight gain is still common. Additionally, approximately 25% of patients do not continue to lose weight as planned [9]. Successful weight loss is defined as more than 50% excess weight loss (EWL). Following gastric bypass surgery, the percent EWL is reported to be between 63% and 78% [10]. However, 15%–20% of patients fail to achieve 50% EWL, according to a systematic review of the effectiveness of surgical treatment for obesity [11].

Prior research suggests that work and family obligations are significant factors contributing to poor dietary adherence after bariatric surgery [12]. Educational programs have not paid enough attention to the patient's family members to improve complications [13, 14]. Health promotion researchers believe that the family plays a crucial role in disease care and treatment [15]. Educating and empowering patients and their families leads to better patient outcomes and increased participation in healthcare programs [16].

The family‐centered empowerment model (FCEM) by Alhani is effective in empowering patients with chronic diseases. In this model, the active involvement of the family is crucial in identifying educational needs since the occurrence of a disease affects not only the individual but also the entire family. It consists of four stages: enhancing knowledge, boosting self‐efficacy, elevating self‐esteem, and assessing. This model has been widely studied, and its positive results are well‐established [17]. However, there is no evidence proving the effectiveness of FCEM for patients undergoing bariatric surgery. Thus, providing education to empower both the patient and family is considered a crucial aspect of nursing care.

Adherence has been defined as the degree to which a patient's voluntary behavior corresponds with the clinical recommendations of healthcare providers and suggests that patients are self‐sufficient individuals who assume an active and voluntary role in defining and achieving goals for their medical treatment [18]. Adherence is an important component of a patient's physical, mental, emotional, and social well‐being that has multiple dimensions. It is particularly crucial for individuals with chronic disease, such as chronic heart failure [19], hypertension, Type 2 diabetes, and maintenance dialysis [20]. Social support is the feeling of being cared for by others and having a support system to rely on. It comes from loved ones, friends, and partners and is related to the frequency of supportive acts [21]. It can shape people's views of themselves and the world [22]. Strong social relationships may have a greater impact on mortality risk than traditional factors such as smoking status or level of physical activity [23]. Having social support can lead to higher life satisfaction and fewer negative emotions [24, 25].

Studies have shown that although bariatric surgery can be successful in reducing the weight of patients, some individuals may not experience weight loss due to various factors. Thus, the aim of this study was to evaluate the effects of two educational methods on adherence and perceived social support in bariatric surgery patients: the FCEM versus the traditional model.

## 2 Materials and Methods

**Figure 1:**
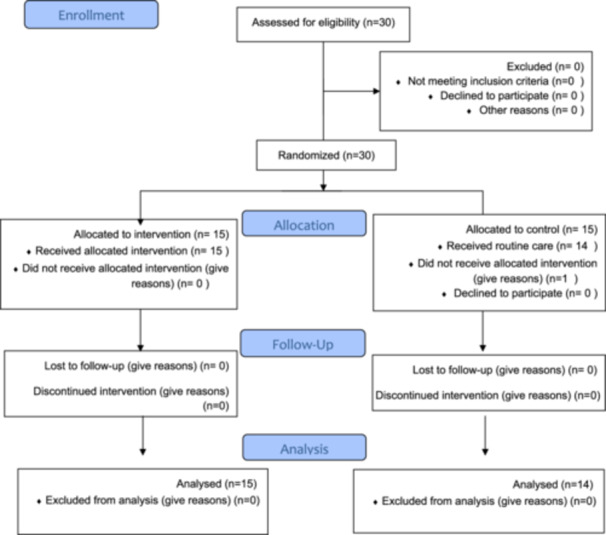
CONSORT flow chart of the participants.

This randomized controlled trial study was conducted in one of the hospitals of Shiraz University of Medical Sciences, Elective Bariatric Surgery Center between April and July 2020 on all bariatric surgery patients. Due to the limited number of bariatric surgeries and considering the time period, all individuals who referred to the center within a 6‐month period who met the study inclusion criteria were considered as the sample size. Finally, 30 people entered the study and were assigned to two control and intervention groups in a permutation block (Figure 1: CONSORT flow chart).

### 2.1 Inclusion Criteria Comprised

1. Willingness to provide written informed consent for research participation,
2. Candidacy for bariatric surgery (gastric bypass, sleeve gastrectomy, mini gastric bypass, or equivalent procedures),
3. Designation as the patient's primary caregiver,
4. Possession of a minimum educational qualification by the primary caregiver (high school diploma or equivalent).

### 2.2 Exclusion Criteria Included

1. Diagnosis of endocrine disorders or other medical conditions causing treatable obesity,
2. Presence of severe, diagnosed psychiatric disorders (psychotic disorders, bipolar disorder, or major depressive disorder with active suicidal ideation and others).
3. Active alcohol or substance dependence,
4. Unwillingness to continue participation in the study.

This study employed permuted block randomization to ensure balanced allocation of participants between the two groups. A total of 30 participants were randomly assigned to either Group A (*n* = 15) or Group B (*n* = 15) using blocks of size 6. The randomization sequence was computer‐generated using the Random Allocation Software to minimize selection bias. Each block contained an equal distribution of participants in a randomly permuted order (e.g., AABBAA, BBAAAB). Due to the nature of the study and the inability to blind the researchers, blinding was only done for the data processor and the study is single‐blind.

In the present study, intervention protocol FCEM was conducted for the patient and his/her caregiver, and a demographic information questionnaire was completed for both. However, the study questionnaires were completed only for the patient.

### 2.3 Instruments

Four paper‐based questionnaires were used for data collection from participants before and after the intervention. After the questionnaires were filled out by the participants, they were evaluated by the researcher to ensure that patients have responded to all items. Questionnaires 2–4 were completed only for the patient after intervention.

Several laboratory parameters consisted fasting blood sugar (FBS), triglycerides (TGs), and total cholesterol levels were measured post‐intervention and compared between the two groups.

Several laboratory parameters consisted fasting blood sugar (FBS), triglycerides (TGs), and total cholesterol levels were measured post‐intervention and compared between the two groups.

1. **Demographic questionnaire:**
  A self‐reported form collecting general information of the participants (the patient and her/his caregiver) including age, gender, education, marital status, job, affliction ‎to a specific physical or psychological illness, and drug consumption with informed consent documentation.
2. **Bariatric surgery self‐management behaviors questionnaire (BSSQ) (Welch et al. 2007):**
  Welch et al. [26] developed the BSSQ to measure the patient's self‐management behaviors over the previous week in a brief, practical, and behavioral manner [26]. The original version of BSSQ consists of 33 items and has seven behavioral domains. Items in the BSSQ are scored using Likert scale with responses ranging from “never,” “sometimes,” and “always.” Subscales and total scores are converted to a 33–99 range, with higher scores indicating better adherence. Psychometric analysis results for original version of BSSQ has shown acceptable results. internal reliability of the seven subscales (0.63 ≤ *α* ≤ 0.83) and the overall test (*α* = 0.83) were acceptable. Two‐week test–retest reliabilities (ICC) for the seven subscales (0.46 ≤ ICC ≤ 0.72) and total score (ICC = 0.71) were acceptable. Also, BSSQ construct validity was examined and confirmed [26].
  In the present study, the Persian version of the questionnaire was used. In Sobhani et al.'s [27] study, by using the factor analysis method, 6 subscales for BSSQ were obtained (30 items) in the Iranian population (eating behaviors, fluid intake, vitamin and mineral supplement intake, fruit, vegetable, whole grains, and protein intake, physical activity and dumping syndrome management). Items in the BSSQ are scored using Likert scale with responses ranging from “never,” “sometimes,” and “always.” Subscales and total scores are converted to a 30–90 range, with higher scores indicating better adherence. Psychometric analysis results for Persian version of BSSQ has shown acceptable results. the specific values of each of the six subscales were 61.54% of the variance of self‐management behaviors after bariatric surgery. The reliability coefficient for this questionnaire (Cronbach's alpha = 0.90) was obtained. Using the split‐correlation method, the two‐part correlation was 0.78, indicating the desired reliability of the self‐management behavior questionnaire after bariatric surgery [27].
3. **General Adherence Scale (GAS) and Specific Adherence Scale (SAS) (Hayes et al. [** 28 **]):**
  Hayes et al. [28] developed general and SASs for evaluating chronic disease adherence. The GAS consists of five items, which is used to assess the overall tendency of patients with chronic diseases to comply with doctor's recommendations in the last 4 weeks. The response format of GAS is a 6‐point Likert scale, ranging from “*None of the time*” (0) to “*All of the time*” (5). The total score of the scale is from 5 to 25. The GAS scale has one dimension, and its internal consistency reliability is acceptable (*α* = 0.78). SAS measures compliance with the recommendations for a disease, and has 10 items on a Likert scale of six degrees (score range between 0 and 50) of internal consistency obtained for it within acceptable limits (*α* = 0.73). The reliability of this scales as determined by Hayes and colleagues [28] based on the correlation of the test‐retest interval of 2 years is satisfactory (SAS = 0.55 and GAS = 0.60).
  In the present study, the Persian version of the questionnaire was used and the scale was adjusted for measures adherence of patients suffering from obesity advice on diet, supplements and lifestyle. The GAS and the specialized adherence scale obtained alpha coefficients as 0.76 and 0.87, respectively, in this study.
4. **Multidimensional Scale of Perceived Social Support (MSPSS) (Zimet et al. [** 29 **]):**
  For the evaluation of Perceived Social Support, the Multidimensional Scale of Perceived Social Support Questionnaire (MSPSS) was used [29]. In this questionnaire, subjects are asked how they perceive social support from relevant sources. The original version of ***MSPSS*** consists of 12 items and has three dimensions: family (four items), friends (four items), and other significant ones (four items). Items in the MSPSS are scored using Likert scale with responses ranging from 1 = *strongly disagree* and 5 = *strongly agree* (total score range between 12 and 60). Higher scores indicate greater perceptions of social support.
*The original version of the tool has demonstrated robust psychometric properties, supported by validation in numerous studies* [29, 30]. Psychometric analysis results for Persian version of MSPSS has shown acceptable results. Cronbach's alpha coefficients for the full scale and its three subscales; family, other significant ones, and friends' support were calculated as 0.91, 0.87, 0.83, and 0.89, respectively, confirming the internal consistency of the Persian version of the scale [31, 32].

### 2.4 Intervention Group

FCEM intervention was held at Ghadir Child and Mother Hospital, Shiraz, Iran, after obtaining the required permissions. FCEM was used as the basis for the intervention. Patients and their families are encouraged by this model to improve their health. In this model, four steps are involved: (a) team discussion, (b) self‐efficacy, (c) education, and (d) process and outcome assessments [33]. The FCEM was performed based on the steps described in Table 1 (based on the standardized protocols and guideline), and was conducted through eight educational sessions (two sessions per week), each lasting 45–60 min (lectures, group discussions, questions and answers, examples, demonstrations, and brainstorming‎), for both the patient and their caregiver. The first two sessions were held preoperatively, while the remaining sessions took place over the 3 weeks following surgery. In‐person sessions were held face‐to‐face at the healthcare facility with both the patient and caregiver in attendance. The training was delivered by the research team (a nurse educator and a clinical psychologist and et al.).

**Table 1:** Topics and content presented in the empowerment sessions.

	Topics	Content of sessions
1	Perceived threat	To familiarize the participants with the problems and burdens caregivers face due to diseases mismanaged and non‐compliance with self‐care, patients were visited twice, and their caregivers were interviewed.
2	Self‐efficacy	Researcher supervised the exchange of experiences between patients, who gave concrete examples of their conditions by discussing, and exchanging them with each other.
		As part of self‐care behaviors, patients get up, take a bath, and exercise. Kegel exercises were demonstrated step‐by‐step by the researcher (and then practiced by patients).
3	Self‐esteem	Researchers have observed the patient and caregiver teaching 1–2 other patients what they have learned.
		Patients and caregivers should be encouraged to educate each other.
4	Evaluation	Each session began with an evaluation of the stages of the family‐centered empowerment model (perceived threat, self‐efficacy, self‐esteem). During the intervention process, questions about the illness and care learned during the training classes were asked to assess recall of the content of previous sessions. During the week following the training, two phone calls were made.

The FCEM was performed based on one of the most significant cultural models from Iran and was created with the intention of empowering patients and their families. ‎To create deeper learning, we provided appropriate images, slides, and educational videos along with the practical training. Researchers asked the caregivers to practice training and then identify their limitations and strengths during the training presentation. In addition, caregivers were encouraged to practice if deficiencies existed. To maintain a continuous relationship with caregivers, we implemented a follow‐up program. To facilitate communication, the researchers provided the participants with the contact information of one of the researchers, so that they could call if they had questions or needed assistance.

### 2.5 Control Group

In this patient group, the standard hospital education protocol was administered, with no additional instructional interventions provided. Upon study completion, the educational materials delivered to the intervention group were compiled into a digital manual booklet and distributed immediately after the intervention period and completing the questionnaires to participants in the control group.

### 2.6 Post‐Test Intervention

Questionnaire completion and measurements of anthropometric and laboratory factors were conducted 1 week after the final educational session (1‐month post‐surgery) by intervention and control group.

### 2.7 Ethical Considerations

The stages of this study implementation were approved by the ethics committee of Shiraz University of Medical Sciences (No.: IR.SUMS.REC.1398.515) and registered on Iranian randomization clinical trial with No. IRCT, IRCT20180523039802N3.‎ Registered on December 13, 2020. At the outset, the study objectives were explained to the patients who participated in the study, and written informed consent was obtained. Additionally, the participants were assured of the confidentiality of their information and the voluntary nature of their participation.

### 2.8 Statistical Analyses

Statistical analysis was performed using SPSS version 21. Descriptive statistics (mean, standard deviation, frequency, and percentage) were used to summarize demographic and baseline characteristics. For inferential analysis, independent *t*‐tests were used to compare between‐group differences, and paired *t*‐tests were applied to evaluate within‐group changes before and after the intervention. Chi‐square tests were used to analyze associations between categorical variables. A significance level of 0.05 was considered for all statistical tests.

## 3 Results

According to the findings, the mean (SD) age of the participants in the intervention group was 47.27 (14.49) years, ranging from 25 to 77 years. In contrast, the control group had a mean (SD) age of 40 (10.66) years, with an age range of 17–54 years. The independent *t*‐test results did not show a significant difference in the mean scores of ages between the two groups (*p* = 0.138) (Table 2).

**Table 2:** Baseline demographic characteristics of Intervention and Control groups.

Participants	Variable	Category	Intervention (*n* = 15)	Control (*n* = 14)	Total (*n* = 29)	*p* value*
Patients	Gender	Female	7 (46.7%)	9 (64.3%)	16 (55.2%)	0.340
		Male	8 (53.3%)	5 (35.7%)	13 (44.8%)	
	Education	Lower than diploma	3 (20%)	2 (14.3%)	5 (17.2%)	1
		Diploma	4 (26.7%)	3 (21.4%)	7 (24.1%)	
		Higher than diploma	8 (53.3%)	9 (64.3%)	17 (58.6%)	
	Marital status	Single	4 (26.7%)	7 (50%)	11 (37.9%)	0.196
		Married	11 (73.3%)	7 (50%)	18 (62.1%)	
	Job	Homemaker	6 (40.0%)	3 (21.4%)	9 (32.1%)	0.163
		Retired	2 (13.3%)	0 (0.0%)	1 (3.6%)	
		Employed	7 (46.7%)	11 (78.6%)	18 (64.3%)	
	Affliction to illness	Yes	4 (26.7%)	3 (21.4%)	7 (24.1%)	1
		No	11 (73.3%)	11 (78.6%)	22 (75.9%)	
	Drug consumption	Yes	9 (60.0%)	7 (50.0%)	16 (55.2%)	0.588
No		6 (40%)	7 (50.0%)	13 (44.8%)		

Caregivers	Gender	Female	10 (66.7%)	8 (51.1%)	18 (62.1%)	0.597
		Male	5 (33.3%)	6 (42.9%)	11 (37.9%)	
	Education	Lower than diploma	1 (6.7%)	2 (14.3%)	3 (10.3%)	0.868
		Diploma	10 (66.7%)	8 (57.1%)	18 (62.1%)	
		Higher than diploma	4 (26.7%)	4 (28.6%)	8 (27.6%)	
	Marital status	Single	10 (66.7%)	8 (57.1%)	18 (62.1%)	0.597
		Married	5 (33.3%)	6 (42.9%)	11 (37.9%)	
	Job	Homemaker	7 (46.7)	7 (50%)	14 (48.3%)	0.501
		Retired	5 (33.3%)	2 (14.3%)	7 (24.1%)	
		Employed	3 (20%)	5 (35.7%)	8 (27.6%)	

* Chi‐squared test.

According to the findings, the mean (SD) of the height in the intervention group was 162.57 (7.52) cm, and in contrast group was 169.57 (5.46) cm, that the independent *t*‐test results showed a significant difference between the two groups (*p* = 0.009), the control group were taller than the experimental group. Additional anthropometric variables are summarized in Table 3.

**Table 3:** Comparison of anthropometric characteristics among participants in the Control and Intervention groups.

Variable	Time	Intervention (*n* = 15) Mean ± SD	Control (*n* = 14) Mean ± SD	*p* value*
Weight	Before	114.00 ± 20.15	110.29 ± 9.10	0.533
	After	87.20 ± 8.39	92.77 ± 9.54	0.112
*p* value**		*p* ≤ 0.0001	*p* ≤ 0.0001	
BMI	Before	42.06 ± 4.43	43.53 ± 2.95	0.306
	After	39.63 ± 3.27	40.30 ± 2.73	0.563
*p* value**		0.057	*p* ≤ 0.0001	
WC^a^	Before	104.51 ± 7.46	106.74 ± 11.31	0.533
	After	92.30 ± 8.50	99.80 ± 9.46	0.032
*p* value**		*p* ≤ 0.0001	0.017	
HC^b^	Before	118.93 ± 11.24	114.79 ± 12.58	0.358
	After	97.71 ± 5.6	102.44 ± 6.14	0.039
*p* value**		*p* ≤ 0.0001	*p* ≤ 0.0001	

*Note:* WC^a^ = waist circumference, HC^b^ = hip circumference.

* Independent *t*‐test.

** Paired *t*‐test.

A comparison of post‐intervention laboratory values revealed no significant intergroup differences in FBS (*p* = 0.419), TGs (*p* = 0.278), and total cholesterol levels (*p* = 0.289), as assessed by an “independent *t*‐test” (Table 4).

**Table 4:** Comparison of laboratory values among participants in the Control and Intervention groups.

Variable	Mean ± SD		Mean difference	*p* value*
	Intervention (*n* = 15)	Control (*n* = 14)	Group1–Group2	
TG	159.40 ± 21.91	150.36 ± 22.09	9.042	0.278
CHOL	167.20 ± 22.97	272.93 ± 378.74	−105.729	0.289
LDL	83.33 ± 9.52	89.14 ± 19.70	−5.809	0.330
HDL	64.87 ± 11.52	58.50 ± 12.68	6.367	0.168
FBS	97.07 ± 27.90	103.29 ± 5.13	−6.219	0.419

Abbreviations: CHOL, total cholesterol; FBS, fasting blood sugar; HDL, high‐density lipoprotein; LDL, low‐density lipoprotein; TG, triglycerides.

* Independent *t*‐test.

The results showed that there was no statistically significant difference in Self‐management behavior (*p* = 0.255), and General Adherence (*p* = 0.170), between the two groups after the intervention, but there was a significant difference in total Perceived Social Support (*p* = 0.015) and tow subscales; family (*p* = 0.006), and friends' support (*p* = 0.037) between the two groups after the intervention (Table 5).

**Table 5:** Comparison of Self‐management behavior, Adherence, and Social support between Intervention and Control groups.

Variable	Category	Intervention group (mean ± SD)	Control group (mean ± SD)	*p* value*
Self‐management behavior		41.80 ± 7.79	45.14 ± 7.66	0.255
Adherence	General compliance	20.07 ± 2.79	18.28 ± 3.95	0.170
	Specific compliance	47.80 ± 7.09	42.50 ± 6.48	0.046
Social support	Family' support	18.33 ± 0.97	17.00 ± 1.83	0.006
	Friends' support	17.93 ± 1.38	16.78 ± 2.39	0.037
	Significant ones' support	17.80 ± 1.74	17.21 ± 1.67	0.87
	Total social support	54.06 ± 2.89	51.00 ± 5.05	0.015

* Independent *t*‐test.

## 4 Discussion

In this randomized clinical trial targeting adherence and perceived social support, we found no statistically significant difference between the two groups in terms of adherence and perceived social support. Although patients who were randomized to use FCEM showed more adherence than the control group, the mean of adherence did not differ between the study groups.

Our search did not find any studies that evaluated the FCEM model for patients undergoing bariatric surgery, which shows the novelty of our current study. The most relevant articles analyze and summarize the most crucial findings and may also help identify potential areas for future research. Furthermore, it could help to identify potential areas for further research.

Based on our findings, FCEM does not significantly impact general adherence to bariatric surgery, however there was a significant difference in Specific compliance between the two groups after the intervention. These results were not consistent in the same with line that of Spetz et al. [13], but the results were consistent with those of Galyean et al. [34]. The difference in these results can be attributed to differences in the type of intervention and more adherence to the drug. Also, according to this study, long‐term postoperative follow‐up can lead to the patients' general adherence to treatment.

Our study showed that the intervention group patients lost weight significantly after surgery. The results obtained were consistent with those of Tan et al.'s [35] cohort study findings Hjelmesth et al.'s [36]; they found similar results, even though the type of intervention was different. In contrast, Ferber et al. [37] reported different results. There may be cultural and behavioral differences between family members that are responsible for this. Different cultures have different norms and behaviors when it comes to family relationships, and these can affect how family members interact with one another and how they respond to certain situations. Therefore, it is possible that Ferber et al.'s [37] results do not represent all families.

In addition, our study found a significant difference in the mean BMI before and after the intervention. Another study in Turkey supports this finding [38]. Nevertheless, Nancarrow et al.'s [39] study and Boon et al.'s [40] research showed conflicting results. There are some explanations for this contrast. First, the difference in results could be due to different follow‐up times of the patients. Varied ‎follow‐up times can cause variations in the patients' responses to treatment, symptom severity, ‎and complications. Therefore, the length of patient follow‐up might have influenced the ‎outcome of Boon et al.'s [40] study. Second, other factors such as self‐esteem, self‐efficacy and other related psychological factors may be effective in reducing the index other than family. These psychological factors help individuals make healthier choices in terms of their diet and exercise. Furthermore, they can also help individuals develop a positive attitude towards their body image, which can further contribute to reducing their body mass index [41].

Our study found that total perceived social support and and tow subscales; family and friends' support in the intervention group was higher than the control group, but the mean of perceived social support in significant ones' support was not statistically significant between the two study groups. The literature does not contain any studies that look at how well FCEM works with perceived social support. As so, this study will be recognized as a pioneering study in the literature. The results of this investigation can help guide future research.‎

Additionally, weight loss between the two groups showed a significant difference pre‐ and post‐ intervention. A different study conducted by Norwegian researchers reported opposite findings [36]. According to the present study, the differences may be due to psychological interventions made in the previous study. After bariatric surgery, psychological factors play a crucial role in maintaining weight and health. Psychological interventions can help patients to develop healthy eating habits and build self‐efficacy, which are essential for long‐term success. Therefore, it is important to provide psychological support after bariatric surgery.

This study had some limitations. First, this is the first study to evaluate the effect of FCEM on perceived social support and adherence in patients undergoing bariatric surgery, resulting in limited comparisons. Studies should be conducted on different statistical communities and in groups with larger sample sizes to evaluate the effectiveness of this model on bariatric surgery patients. Comparing this model with psychological models is also suggested. Second, the duration of follow‐up of patients was low due to time constraints; longer patient follow‐ups may result in different outcomes. Patients were followed up for a short period. Future studies should, therefore, follow up the patients at longer intervals. Results may differ if a patient is followed up for a longer durations. Finally, this study did not consider psychological factors. Future studies are suggested to investigate the effects of this model on various psychological factors.

## 5 Conclusion

Family‐centered models provide an opportunity to empower family members to become active participants in the patient's health and perceived social support. The model could provide an opportunity for the patients to develop health their habits, such as physical activity, which would help to maintain their weight loss. However, it has not been proven that the family‐centered model is effective in improving self‐management behavior and general adherence.

## Author Contributions

**Mahboobeh Hosseinimoghadam** and **Zinat Mohebbi:** conceptualization, data curation, formal analysis, investigation, methodology, project administration, resources, software, supervision, validation, visualization, writing, review and editing. **Sina Ghanbarzadeh** and **Zahra Sobhani:** conceptualization, data curation, formal analysis, funding acquisition, investigation, methodology, project administration, resources, supervision, writing original draft, writing, review and editing. **Masood Amini** and **Amirali Alizadeh:** data curation, investigation, supervision, writing, review and editing. All authors contributed to the critical revision of the manuscript. All authors approved the final submission of the manuscript.

## Ethics Statement

Ethical matters, e.g., plagiarism, informed consent, misconduct, data fabrication and/or falsification, double publication and/or submission, redundancy, etc., have been totally observed by the authors. Also, ethics committee of Shiraz University of Medical Sciences of Medical Science has approved the research with ethical No.: IR.SUMS.REC.1398.515.

## Consent

All the participants received verbal explanation about the study objectives and procedures and then signed written informed consents for taking part in the study. The participants were also reassured about the anonymity and confidentiality of their information.

## Conflicts of Interest

The authors declare no conflicts of interest.

## Transparency Statement

The lead author, Zinat Mohebbi, affirms that this manuscript is an honest, accurate, and transparent account of the study being reported; that no important aspects of the study have been omitted; and that any discrepancies from the study as planned (and, if relevant, registered) have been explained.

## Data Availability

The datasets supporting the conclusion of this study are available upon reasonable request from the corresponding author.
